# Differences in outcomes regarding quality and speed between male and female endoscopists in a virtual gastroscopy course

**DOI:** 10.3389/fsurg.2026.1854687

**Published:** 2026-08-12

**Authors:** Nathalie Young, Peter Elbe, Charlotte Höög, Ninos Oussi, Fredrik Swahn, Magnus Kjellman, Gabriel Sandblom, Lars Enochsson

**Affiliations:** 1 Department of Clinical Science and Education, Södersjukhuset Karolinska Institute, Stockholm, Sweden; 2 Department of Surgery, Södersjukhuset, Stockholm, Sweden; 3 Department of Upper Abdominal Diseases, Karolinska University Hospital, Stockholm, Sweden; 4 Division of Surgery, CLINTEC, Karolinska Institutet, Stockholm, Sweden; 5 Department of Medicine Huddinge, Karolinska Institute, Stockholm, Sweden; 6 Centre for Clinical Research, Region Sörmland, Uppsala University, Eskilstuna, Sweden; 7 Department of Molecular Medicine and Surgery, Karolinska Institutet, Stockholm, Sweden; 8 Department of Breast Endocrine Tumours and Sarcoma, Karolinska University Hospital Stockholm, Sweden; 9 Department of Diagnostics and Intervention Surgery, Umeå University, Umeå, Sweden; 10 Division of Orthopaedics and Biotechnology, CLINTEC, Karolinska Institutet, Stockholm, Sweden

**Keywords:** endoscopy simulation training, gastroscopy, gender differences, surgical education, virtual reality simulations

## Abstract

**Introduction:**

While simulator training is known to enhance endoscopic proficiency, gender differences in learning styles and outcomes remain underexplored in upper gastrointestinal endoscopy. This study aimed to evaluate improvements in technical skills in a simulated environment, highlight gender differences in performance, and assess the perceived value of the course among surgical and gastroenterology trainees.

**Materials and methods:**

In this observational pre- and post-course study, surgical and gastroenterology trainees (*n* = 19, 10 females, 9 males) participated in a four-day introductory endoscopy course. The curriculum combined theoretical instruction with supervised simulator training using the GI-Mentor II™ (gastroscopy case and Endo Bubble exercise 1) and the Thompson Endoscopic Skills Trainer (TEST) from EndoSim™ (polyp snaring exercise). Pre- and post-course performance was assessed based on completion times, screening efficiency, and the extent of mucosa inspected. Between-group comparisons were performed on the entire cohort as well as paired comparisons stratified by gender. Two questionnaires evaluated baseline characteristics and perceived course value.

**Results:**

Overall, trainees demonstrated significant improvements in technical skills following the course. No significant differences were observed between female and male participants at baseline. Post-course, female participants were significantly faster in reaching the descending part of duodenum (*p* = 0.0184). Male participants showed greater gains in speed-related tasks, while female participants demonstrated larger gains in quality-related performance metrics (screening efficiency and mucosal inspection quality). The course was perceived as highly valuable, with most participants (89.5%) reporting increased confidence in performing endoscopy in a clinical setting.

**Conclusions:**

The course significantly improved technical skills and performance quality in simulator-based endoscopy. The observed differences in improvement patterns between participants highlight the potential value of adapting training to individual learning profiles rather than broad categorisations. Such targeted approaches may help optimise endoscopic education and ultimately enhance patient safety.

## Introduction

It is well established that simulation-based training improves outcomes in both gallstone surgery (1–4) and endoscopy (5, 6), reducing error rates and enhancing procedural proficiency in a risk-free environment. Proficiency-based virtual reality (VR) training significantly reduces the risk of complications during early laparoscopic procedures (4), and comparable benefits extend to endoscopy, where simulation facilitates efficient screening and thorough mucosal inspection (5). Despite previously documented gender-based differences in simulator performance, particularly the tendency for male participants and individuals with extensive video-gaming experience to complete tasks more rapidly (7–12), the influence of participant gender on course perception and the pedagogical implications for future course design remain largely unexplored. Notably, with additional practice, such as through simulation-based mastery learning, female participants and individuals without prior gaming experience can achieve outcomes comparable to, or even surpassing, those of their peers (2, 6, 13, 14). These findings align with a recent Swedish registry-based study on cholecystectomy, which reported that although female surgeons had longer operative times than their male counterparts, their procedures resulted in significantly lower complication rates (15).

Basic Interactive Endoscopy in a Simulator Environment is a course offered at the Centre for Advanced Medical Simulation and Training (CAMST) at Karolinska University Hospital, Stockholm, Sweden. Organised by a national faculty appointed by the Swedish Surgical Association and the Swedish Society of Gastroenterology, this course has been offered to surgical and gastroenterology trainees since 2015; however, it has not yet been formally validated. The rationale behind its inception was to bridge an educational gap and progress towards a national standard, specifically targeting trainees in surgery and gastroenterology, as these are the clinical specialists who primarily perform endoscopy in Sweden. Through a combination of practical training sessions and theoretical content, the course aims to provide participants with a solid foundation for recognising and treating the most common conditions when performing endoscopy on their first patients. As with many other courses in surgery and endoscopy, a substantial proportion of the curriculum is devoted to simulation-based skills training, alongside the diagnosis and management of gastrointestinal diseases.

Beyond this course, there is currently an absence of nationally standardised training for aspiring endoscopists in Sweden. Training is generally provided locally at individual clinics without a predefined curriculum or routine access to endoscopy simulators prior to clinical practice. This absence of a structured educational pathway may impede skill development, contribute to unnecessary patient discomfort, and potentially compromise patient safety.

The primary aim of this study was to evaluate the extent to which trainees participating in the Basic Interactive Endoscopy in a Simulator Environment course demonstrated improvements in technical performance during endoscopic simulator exercises. A secondary aim was to examine whether these course outcomes differed according to participant gender. Importantly, in this context, gender is viewed as a proxy for underlying individual factors, such as previous visuospatial gaming experience or anatomical variations like hand size. The objective of exploring these differences is not to reinforce stereotypes, but rather to inform the design of future simulator platforms and curricula. By identifying distinct learning trajectories, training can be tailored to support precision and rigorous mucosal inspection in trainees prone to prioritizing rapidity, while concurrently encouraging pace and efficiency in those who adopt a more overly cautious approach.

## Materials and methods

In this observational pre- and post-intervention study, surgical and gastroenterology trainees (*n* = 19; 10 females, 9 males) with comparable baseline knowledge and similar prior endoscopic experience were included. Participation was voluntary and the assessment was not blinded. At baseline, participants had limited practical experience, having performed fewer than 10 complete gastroscopies or only partial procedures under supervision. Five participants reported prior video-gaming experience; of these, two had engaged in visuospatial gaming within the preceding week (for one and three hours, respectively), whereas the remaining three had not played since childhood.

**Figure 1:**
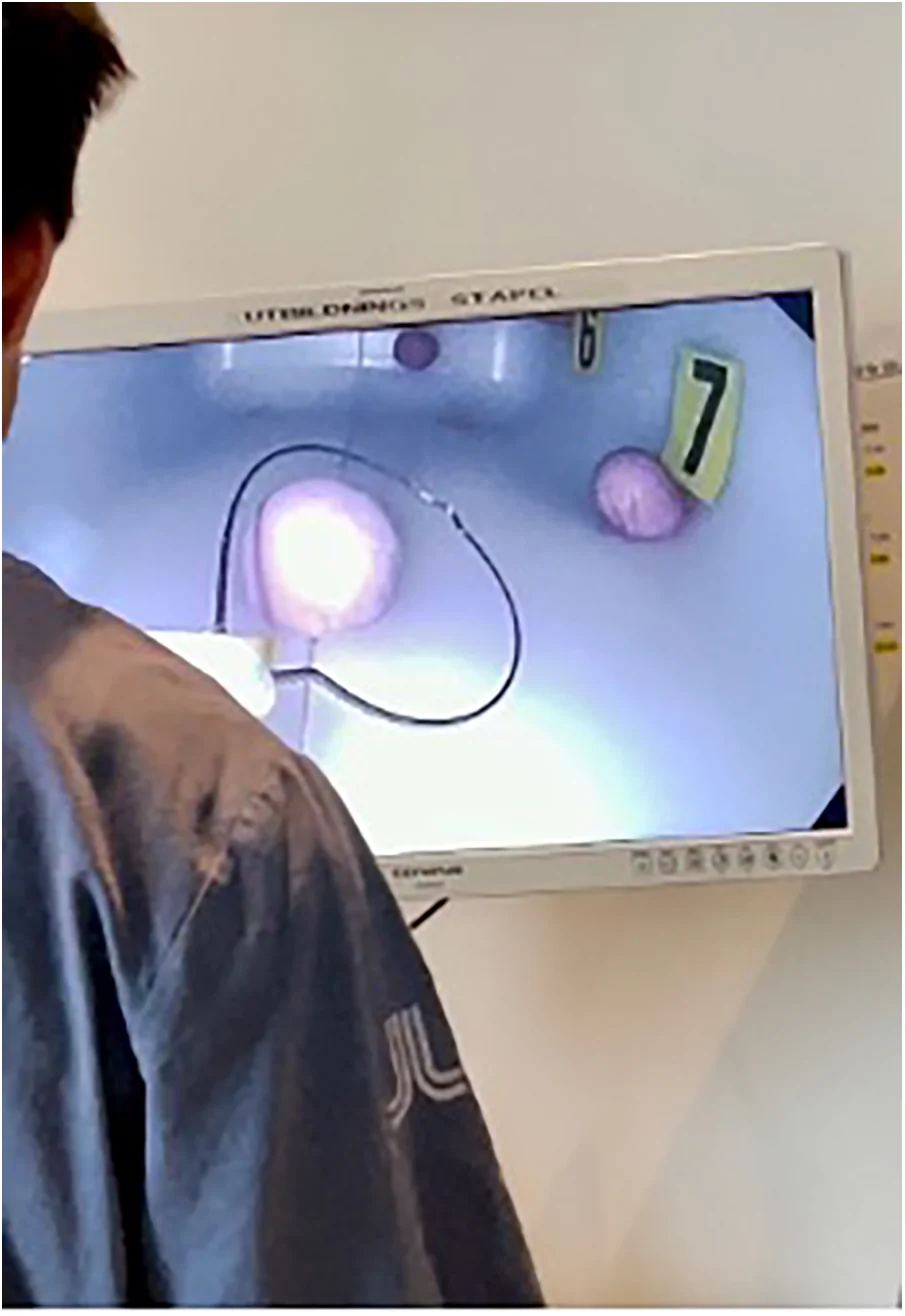
One of the trainees performing in the thompson endoscopic skills trainer (polyp snaring exercise).

The four-day course integrated theoretical instruction with supervised, simulator-based training using the GI-Mentor II^TM^ (virtual gastroscopy case and Endo Bubble exercise 1) and the Thompson Endoscopic Skills Trainer (TEST) from EndoSim^TM^ (polyp-snaring exercise). The course was delivered at the Centre for Advanced Medical Simulation and Training (CAMST) by experienced endoscopists acting as proctors. In the polyp-snaring task, participants were required to sequentially retrieve nine plastic polyps. A research assistant assessed each snare attempt for approval before the participant was allowed to proceed, and the total completion time was recorded (Figure 1).

### Pre- and post-course performance on the GI-mentor II^TM^ was evaluated using the following simulator-generated metrics in the virtual gastroscopy case

- Screening efficiency (%) was defined as the simulator-generated composite score reflecting the efficiency of mucosal screening, incorporating the time spent with clear luminal view and systematic inspection pattern.
- Mucosal inspection quality (%) (also referred to as percentage of mucosal surface examined) was defined as the percentage of the total gastric mucosal surface visualised during the procedure, calculated by the simulator's 3D surface coverage algorithm.
- Time to reach the descending part of duodenum was defined as the time in seconds from endoscope insertion until the tip of the scope reached the second portion of the duodenum (D2), as automatically registered by the simulator based on anatomical landmarks.

These metrics have been used and reported in previous validation and training studies using the GI-Mentor II^TM^ gastroscopy module. The Endo Bubble 1 exercise is a validated psychomotor task in the GI-Mentor II^TM^. The cyberscope module is designed to train hand-eye coordination and fine motor control. Participants were required to sequentially pop 20 virtual balloons using the endoscope while avoiding wall collisions. Performance was measured by the number of sequential balloon pops (out of 20), successful balloon pops ratio (%), number of wall hits, and total completion time. This exercise has been used in previous studies to assess basic endoscopic navigation skills on the GI-Mentor II^TM^ (10, 16–18).

Pre- and post-course performance was evaluated using tempo-related metrics (time required to complete each exercise) and quality-related metrics (screening efficiency and extent of mucosal inspection). Data were obtained through a combination of manual timing and simulator-generated outputs. Additionally, two Likert-scale questionnaires were administered to assess baseline characteristics and perceived course value.

### Statistical analyses

Changes in continuous performance parameters (total time in the Thompson Endoscopic Skills Trainer, total time for the Endo Bubble 1 exercise as well as the virtual gastroscopy parameters: efficiency of screening, mucosa inspected, time to reach the descending part of duodenum, and total time) between pre- and post-course assessments were analysed using the paired samples t-test or the Wilcoxon Signed-Rank test, as appropriate. Between-group comparisons evaluating baseline and post-course differences between males and females were performed using the Mann–Whitney U-test. Furthermore, to assess sex-specific progression, intra-group changes (pre- vs. post-course) were analysed separately for males and females using the Wilcoxon Signed-Rank test. A two-sided *p*-value <0.05 was considered statistically significant.

The study was approved by the Swedish Ethical Review Authority (2020-04265). All study participants signed informed consent, and participation was entirely voluntary.

## Results

Overall, trainees demonstrated significant improvements in technical skills, on the Thompson Endoscopic Skills Trainer (polyp snaring exercise) and the Endo Bubble 1 exercise, as well as in quality-oriented metrics, such as the percentage of mucosa inspected and efficiency of screening in the virtual gastroscopy case (GI-Mentor II^TM^) (Table 1). However, no significant overall improvements were observed regarding the time to reach the descending part of duodenum or total time in virtual gastroscopy (Table 1). When comparing males and females both pre- and post-course, no significant differences were observed between the groups, with one exception: the time to reach the descending part of duodenum was significantly shorter for females compared to males post-course (38 vs. 66 s; *p* = 0.0184) (Table 2). Subgroup analyses revealed sex-specific differences in learning progression; females demonstrated statistically significant improvements in efficiency of screening (*p* = 0.0234), mucosa inspected (*p* = 0.0391) and time to reach the descending part of duodenum (*p* = 0.0430) post-course, whereas no such improvements were observed in males (Table 3). Males, however, demonstrated significant speed improvements post-course regarding the Thompson Endoscopic Skills Trainer (polyp snaring exercise) and total time in Endo Bubble 1 (Table 3).

**Table 1:** Pre- and post-course performance on the entire cohort for the thompson endoscopic skills trainer polyp-snaring exercise, endo bubble 1 and virtual gastroscopy exercises (median and IQR).

		Pre-course		Post-course		*p*
Simulation	Exercises	Median	IQR	Median	IQR	
	Polyp snaring exercise (sec)	500	341–886	360	283–466	**0**.**0003**
	Total time Endo Bubble 1 (sec)	189	144–253	117	98–180	**0**.**0032**
Virtual gastroscopy	Efficiency of screening (%)	87	66–90	91	87–94	**0**.**0053**
	Mucosa inspected (%)	85	75–88	90	84–93	**0**.**0064**
	Time to reach the descending part of duodenum (sec)	47	32–74	54	32–68	0.5013
	Total time (sec)	295	229–410	272	226–317	0.5019

The bold values are statistically significant.

**Table 2:** Between-group comparisons of performance parameters between male and female participants pre- as well as post-course. (Median and IQR, *p*-values were calculated using the Mann–Whitney U-test).

Simulation	Exercises	Pre-course				
		Males		Females		*p*
		Median	IQR	Median	IQR	
	Polyp snaring exercise (sec)	759	508–891	377	304–547	0.1107
	Total time Endo Bubble 1 (sec)	218	139–263	183	141–251	0.6534
Virtual gastroscopy	Efficiency of screening (%)	87	61–90	85	66–92	0.7129
	Mucosa inspected (%)	84	77–90	86	74–88	1.0000
	Time to reach the descending part of duodenum (sec)	46	31–73	60	33–76	0.7751
	Total time (sec)	323	247–451	260	194–357	0.2364

Simulation	Exercises	Post-course				
		Males		Females		*p*
		Median	IQR	Median	IQR	
	Polyp snaring exercise (sec)	360	310–509	358	248–458	0.7133
	Total time Endo Bubble 1 (sec)	120	99–147	106	98–185	1.0000
Virtual gastroscopy	Efficiency of screening (%)	90	86–95	92	89–94	0.5922
	Mucosa inspected (%)	92	88–93	87	82–94	0.5041
	Time to reach the descending part of duodenum (sec)	66	45–81	38	31–62	**0**.**0184**
	Total time (sec)	301	244–357	261	221–287	0.1426

The bold values are statistically significant.

**Table 3:** Within-group changes from pre- to post-course results for males and females, respectively. (Median and IQR, *p*-values were calculated using Wilcoxon signed-rank test).

Simulation	Exercises	Males				
		Pre-course		Post-course		*p*
		Median	IQR	Median	IQR	
	Polyp snaring exercise (sec)	759	508–891	360	310–509	**0.0039**
	Total time Endo Bubble 1 (sec)	218	139–263	120	99–147	**0.0195**
Virtual gastroscopy	Efficiency of screening (%)	87	61–90	90	86–95	0.1484
	Mucosa inspected (%)	84	77–90	92	88–93	0.1172
	Time to reach the descending part of duodenum (sec)	46	31–73	66	45–81	0.2656
	Total time (sec)	323	247–451	301	244–357	0.6406

Simulation	Exercises	Females				
		Pre-course		Post-course		*p*
		Median	IQR	Median	IQR	
	Polyp snaring exercise (sec)	377	304–547	358	248–458	0,1055
	Total time Endo Bubble 1 (sec)	183	141–251	106	98–185	0,1309
Virtual gastroscopy	Efficiency of screening (%)	85	66–92	92	89–94	**0,0234**
	Mucosa inspected (%)	86	74–88	87	82–94	**0,0391**
	Time to reach the descending part of duodenum (sec)	60	33–76	38	31–62	**0,0430**
	Total time (sec)	260	194–357	261	221–287	0,6953

The bold values are statistically significant.

The questionnaire revealed that the course was perceived as highly valuable; most participants (89.5%) reported increased confidence in performing endoscopy in a clinical setting.

## Discussion

The introductory endoscopy course resulted in significant improvements in trainees’ technical skills and performance quality in simulator-based exercises, supporting its effectiveness as a preparatory step for clinical practice. When analysing the entire cohort, no statistically significant differences were observed between female and male participants at baseline. Post-course, however, female participants were significantly faster than male participants in reaching the descending part of duodenum (*p* = 0.0184). This may indicate the adoption of a more systematic and efficient endoscope handling technique, potentially reflecting improved planning and scope control rather than a focus on speed alone.

Distinct patterns of improvement were also observed, with male participants showing greater gains in speed-related tasks and female participants demonstrating more pronounced improvements in quality metrics. These patterns align with previous simulation-based research and with findings from a Swedish registry study on cholecystectomy, in which female surgeons had longer operative times but lower complication rates (15). However, direct comparisons between virtual gastroscopy and clinical procedures must be made with caution. A shared theme emerged: female participants tend to complete procedures at a more moderate pace while maintaining a stronger emphasis on quality. Historically, early male advantages in speed and precision have been attributed to greater exposure to video gaming and corresponding enhancements in visuospatial cognitive skills (2, 7, 8). Still, structured simulator training enables female participants and novices without prior gaming experience to achieve performance levels comparable to, or surpassing, those of their peers (2, 6, 13).

Several limitations must be acknowledged. The small sample size limits the generalisability of the findings and reduces statistical power, particularly for detecting interactions between time and gender. Furthermore, interpreting performance differences primarily through the lens of gender carries limitations. Individual factors such as hand size, ergonomic compatibility with the endoscope (19, 20), and prior visuospatial or gaming experience are likely more proximal determinants of learning trajectories.

Recent evidence from simulation-based mastery learning curricula suggests that targeted training can substantially reduce initial performance gaps related to such factors. Future training programmes may therefore benefit from more individualised approaches rather than predetermined gender-based adaptations.

Strengths of this study include the prospective pre- and post-course design, the use of objective performance metrics derived from both validated simulator outputs and manual timing, and the stratification by gender within a cohort of trainees with comparable baseline experience. By utilising paired analyses, all participants served as their own controls. Additionally, the combination of theoretical tutoring and hands-on supervision at a dedicated simulation centre reflects a highly realistic introductory curriculum.

Larger, multi-centre studies with long-term evaluations of clinical skill transfer are warranted to substantiate these findings and explore the underlying mechanisms more comprehensively. Relevant endpoints would include procedural success rates (e.g., adenoma detection rates), reductions in procedure-related complications, and decreased rates of missed diagnosis during the early clinical learning phase.

In conclusion, this structured introductory course significantly improved both technical proficiency and the quality of performance in virtual gastroscopy, effectively bridging the current educational gap in early endoscopy training. The findings suggest that training outcomes may vary between individuals, supporting the value of adapting future curricula to individual learning needs rather than oversimplified categorisations. Such approaches have the potential to optimise safe skills acquisition and ultimately contribute to higher standards of patient care.

## Data Availability

The raw data supporting the conclusions of this article will be made available by the authors, without undue reservation.
